# Underestimated Incidence Rate of Pertussis in the Community: Results from Active Population-Based Surveillance in Yiwu, China

**DOI:** 10.3390/microorganisms12112186

**Published:** 2024-10-30

**Authors:** Hanying Dai, Hanqing He, Juan Xu, Yao Zhu, Tao Fu, Bohan Chen, Jie Li, Yuan Gao, Aiping Qin, Maojun Zhang, Zhujun Shao

**Affiliations:** 1 National Key Laboratory of Intelligent Tracking and Forecasting for Infectious Diseases, National Institute for Communicable Disease Control and Prevention, Chinese Center for Disease Control and Prevention, Beijing 102206, China; hanyingdai0815@163.com (H.D.); xujuan@163.com (J.X.); chenbohan@icdc.cn (B.C.); lijie06@icdc.cn (J.L.); gaoyuan@icdc.cn (Y.G.); qinaiping@icdc.cn (A.Q.); zhangmaojun@icdc.cn (M.Z.); 2 Zhejiang Provincial Center for Disease Control and Prevention, Hangzhou 310000, China; hanqinghe@cdc.zj.cn (H.H.); yzhu@cdc.zj.cn (Y.Z.); 3 Yiwu District Center for Disease Control and Prevention, Jinhua 321000, China; 13600591797@163.com

**Keywords:** pertussis, active surveillance, incidence, hospitalization rate, China

## Abstract

Background: The resurgence of pertussis in China underscores the urgency of active surveillance to complement the passive surveillance system. Methods: Active surveillance for pertussis was conducted from 1 June 2021 to 31 May 2022, at Yiwu, Zhejiang province of China. Patients with suspected pertussis were further confirmed as pertussis cases by PCR and culture. The incidence rate of pertussis in the community was estimated. Results: The overall estimated incidence of pertussis was 108.3 per 100,000 (95% CrI: 91.7–126.4). Children aged 4–5 years had the highest incidence (1154.3 per 100,000 [95% CrI: 817.4–1553.5]), followed by infants aged 1 year (836.1 per 100,000 [95% CrI: 434.0–1308.8]). Infants aged 0–4 months had the highest hospitalization rate among the pertussis patients (>50.0%). Although the incidence was low in elderly aged ≥ 60 years, the hospitalization rate was rather high (6.7%). Conclusion: Active surveillance in this study revealed a higher burden of pertussis in Yiwu, China, compared to passive surveillance. Children aged 4–5 years are the dominant population group at risk of pertussis. Infants aged ≤ 4 months are the most vulnerable pertussis patients that require hospitalization treatment. Our results highlight the urgency of large-scale active surveillance of pertussis in China.

## 1. Introduction

Pertussis, a highly contagious respiratory disease caused by the bacterium *Bordetella pertussis*, remains a substantial challenge to public health worldwide [1,2]. Despite widespread vaccination, the incidence of pertussis has been resurging in many countries lately. Various factors contributing to pertussis resurgence include waning immunity, the evolution of *B. pertussis*, and suboptimal protection at the population level resulting from declining vaccination coverage rates and the absence or low uptake of boosters [2,3,4,5].

In China, reported pertussis cases have increased significantly since 2014, from 0.25 per 100,000 in 2014 to 2.11 per 100,000 in 2019 [6]. The incidence of pertussis reduced to 0.31 per 100,000 in 2020 and 0.67 per 100,000 in 2021 during the COVID-19 pandemic, largely resulting from the intensified implementation of non-pharmaceutical interventions such as face masks, social distancing, travel restrictions, and lockdowns [6,7]. However, with the relief of these restrictions, the pertussis incidence surged to a higher level compared to pre-pandemic (2.69 per 100,000 in 2022 and 2.89 in 2023, respectively) [6]. According to the recent report from the National Notifiable Disease Reporting System of China, a sharp increase in pertussis cases was observed, up to 469,712 cases by September 2024 alone, about a 26-fold increase compared to the same period in 2023 [8].

The immunization program for pertussis in mainland China consists of a series of primary vaccinations at the ages of 3, 4, and 5 months, and a booster at 18 months with acellular pertussis vaccine (DTaP). Unlike in other countries, no subsequent booster vaccinations are scheduled beyond the age of 2 years [9]. Cases in children aged 3–10 years have increased in China over the past years [10,11,12,13]. Despite this increase, according to the recently published national report based on passive surveillance, the highest incidence of pertussis remains in the population of <1 year of age [14]. However, the incidence in children aged > 1 year might be severely underestimated by passive surveillance [15]. To address this drawback, active surveillance for pertussis has been implemented in the United States [16], Australia [17], and several European countries [18]. Passive surveillance relies on healthcare providers to report cases based on routine clinical encounters. The identification of cases was limited by health workers’ inadequate awareness of pertussis, suboptimal standardization of tests in medical institutions, and insufficient coordination in case reporting, resulting in a significant underestimation of the actual incidence rate of pertussis. In contrast, active surveillance is to proactively identify cases through systematic efforts, including enhanced awareness of pertussis, education and training in disease diagnosis, standardized testing for suspected pertussis cases, and strengthened coordination in case reporting. This approach optimizes case detection and reporting, providing a more real estimation of pertussis incidence. In this study, we aimed to estimate the incidence rate of pertussis in Yiwu, Zhejiang province, China, through active population-based surveillance.

## 2. Materials and Methods

**Figure 1:**
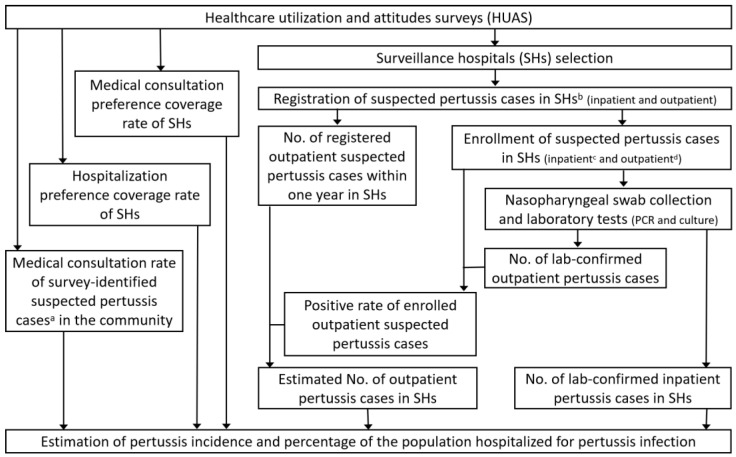
The overall flow of this study (from 1 June 2021 to 31 May 2022 in Yiwu, Zhejiang province). ^a^ The definition of a survey-identified suspected pertussis case was presented in Section 2.2. ^b^ Inclusion and exclusion criteria of suspected pertussis cases in SHs were presented in Section 2.3. ^c^ All inpatient suspected pertussis cases were enrolled during the study period. ^d^ The first 5–10 outpatients with suspected pertussis each week in the SHs were enrolled during the study period. HUAS, healthcare utilization and attitudes survey; PCR, polymerase chain reaction; SHs, surveillance hospitals.

The design of this study comprised three parts: a healthcare utilization and attitude survey (HUAS) in the community, a registration and enrollment of suspected pertussis cases in the surveillance hospitals (SHs), and estimation of pertussis incidence. The study design diagram shown in Figure 1 illustrates how the three parts were interlinked. Briefly, the first part, the HUAS, was designed to obtain data to select SHs with high coverage and extrapolate cases collected at SHs to all hospitals and communities in the city. The second part was to register, enroll, and test the suspected pertussis cases in SHs to identify laboratory-confirmed pertussis cases in SHs. The final part was to apply the data collected in the first two parts and perform calculations regarding the estimation of pertussis incidence and percentage of the population hospitalized for pertussis in the community.

Detailed information regarding the parameters and the calculations was presented in Section 2.2, Section 2.3, Section 2.4 and Section 2.5 and Figure S1A–C. Specifically, the medical consultation preference coverage rate, the hospitalization preference coverage rate of surveillance hospitals (SHs), and the medical consultation rate of survey-identified suspected pertussis cases were obtained through the HUAS. The number of registered and enrolled suspected pertussis cases in SHs and the number of lab-confirmed pertussis cases among enrolled suspected pertussis cases in SHs were collected through the second part.

### 2.1. Study Site and Population

Yiwu, Zhejiang province, was selected as the study site after screening per specific criteria [19], including a strong willingness to participate, capabilities for ongoing surveillance (such as experienced staff, infrastructure for specimen collection, and data security), and the provision of comprehensive healthcare information from hospital information systems. Yiwu is in the central-western part of the Zhejiang province. In 2021, it had a resident population of 1,859,390, served by 24 medical institutions.

In this study, age groups of the population were defined as infants aged < 1 year, young children aged 1 year, 2–3 years, and 4–5 years, school-age children and teenagers aged 6–19 years, adults aged 20–59 years, and elderly people aged ≥ 60 years. For infants aged < 1 year, age groups were further stratified into <3 months, 3 months, 4 months, 5 months, and 6–11 months.

### 2.2. Healthcare Utilization and Attitudes Survey

A healthcare utilization and attitude survey (HUAS) was conducted in Yiwu from 2019 to 2020 to select the participating surveillance hospitals. The methodology and the detailed description of the HUAS have been described previously [19]. In brief, 50 communities from Yiwu were randomly selected through probability proportionate to size sampling, with a quota sampling method subsequently utilized to recruit interviewees within the 50 communities. The surveys were conducted face-to-face by trained staff in every community to ascertain the healthcare-seeking behaviors of the residents.

The medical consultation preference and hospitalization preference coverage rates were calculated by dividing the number of participants who expressed a preference to seek medical consultations or hospitalization, respectively, at a specific hospital by the number of total HUAS respondents, which were computed using Formulas (1) and (2). Hospitals with collectively high coverage rates for medical consultation preference and hospitalization preference were selected as surveillance hospitals. The surveillance hospitals selected were the Yiwu Center Hospital, Yiwu Maternal and Child Healthcare Hospital, and the Fourth Affiliated Hospital of Zhejiang University School of Medicine.

In addition, survey participants who were reported with a cough duration of ≥2 weeks (or infants aged < 1 year regardless of cough duration) and with one or more of the typical pertussis-like symptoms of inspiratory whoop, paroxysmal cough, post-tussive vomiting, worsening coughing at night (for those aged ≥ 1 year), or apnea with or without cyanosis (for those aged < 1 year) within the past month of the survey were identified as ‘suspected pertussis cases’ (we named it survey-identified suspected pertussis cases in later-on data analysis). The medical consultation rate of survey-identified suspected pertussis cases was then calculated by dividing the number of survey-identified suspected pertussis cases that were reported by themselves or their parents to have visited hospitals in the past month by the total number of survey-identified suspected pertussis cases, as shown in Formula (3). Since some suspected pertussis in the community might not visit the hospital, the medical consultation rate of survey-identified suspected cases was used as a reference to estimate the proportion of pertussis patients visiting hospital so that we could make a more precise estimation of pertussis incidence rate in the community.

(1) $$Mpc=\frac{Survey\;participants\;who\;expressed\;a\;preference\;to\;seek\;medical\;consultations\;at\;SHs\;}{Surevey\;participants\;}$$

*Mpc*: medical consultation preference coverage rate of SHs; *SHs*, surveillance hospitals.

(2) $$Hpc=\frac{Survey\;participants\;who\;expressed\;a\;preference\;to\;be\;hospitalized\;at\;SHs\;}{Surevey\;participants\;}$$

*Hpc*: hospitalization preference coverage rate of SHs; *SHs*, surveillance hospitals.

(3) $$Mcs=\frac{Survey-identified\;suspected\;cases\;who\;visited\;hospitals\;in\;the\;past\;month}{\;Survey-identified\;suspected\;cases}$$

*Mcs*: medical consultation rate of survey-identified suspected pertussis cases.

### 2.3. Inclusion and Exclusion Criteria of Suspected Pertussis Cases in SHs

Patients seeking a medical consultation at surveillance hospitals between 1 June 2021 and 31 May 2022 (except during national holidays) were screened for pertussis through the following two steps: Firstly, patients who met both of the following criteria were included: (1) based on the clinical features of suspected pertussis cases in the guidelines for the diagnosis of whooping cough [20,21], patients with International Classification of Diseases, 10th Revision (ICD-10) codes A37, J00-J22, J40-J47, R05, R09.2, P22, P28.2, P28.3, P28.4, and P28.5 as a diagnosis; (2) patients who had a cough duration of ≥2 weeks (or infants aged < 1 year regardless of cough duration) and with one or more of the following typical pertussis-like symptoms: (a) inspiratory whoop, (b) paroxysmal cough, (c) post-tussive vomiting, (d) worsening coughing at night (for those aged ≥ 1 year), or (e) apnea with or without cyanosis (for those aged < 1 year), or alternatively, a physician’s diagnosis of suspected pertussis by overall judgment were also included as a suspected pertussis case. Secondly, patients who met ≥ 1 of the following criteria were excluded: (1) patients diagnosed with gastroesophageal reflux, spastic bronchitis, diagnosed tuberculosis, mycoplasma/chlamydia infection, or chronic sinusitis; (2) adults and adolescents with body temperature ≥ 38.5 °C; and (3) current residential address outside the study area.

All inpatient suspected pertussis cases and the first 5–10 outpatient suspected pertussis cases each week in the three surveillance hospitals were enrolled, and the total number of suspected pertussis cases (including outpatient suspected pertussis cases who were not enrolled) was registered by clinicians at the end of each day. During enrollment, sociodemographic, clinical, and epidemiological information were collected.

### 2.4. Specimen Collection and Laboratory Testing

Nasopharyngeal swabs for pertussis confirmation were collected from those enrolled using culture (Charcoal Agar, Thermo Fisher Scientific, Waltham, MA, USA) and polymerase chain reaction (PCR; triple-channel real-time PCR method; *B. pertussis*, *B. parapertussis*, and *B. holmesii* Detection Kit, MABSKY) methods. The laboratory test procedures followed the World Health Organization’s (WHO) recommended methods [21] and the manufacturer’s instructions. Suspected pertussis cases with positive results of PCR and/or culture were identified as pertussis cases.

The positive rate of enrolled outpatient suspected pertussis cases was calculated by dividing the number of lab-confirmed outpatient pertussis cases by the number of enrolled outpatient suspected pertussis cases as Formula (5).

The estimated number of inpatient pertussis cases in all hospitals of Yiwu(*Eip*) and estimated number of outpatient pertussis cases in all hospitals of Yiwu(*Eop*) were calculated using Formulas (4) and (6) by dividing inpatient or outpatient pertussis cases in SHs by respective coverage rate (i.e., hospitalization preference coverage rate(*Hpc*) for *Eip* or medical consultation preference coverage rate(*Mpc*) for *Eop*). As not all outpatient suspected cases in SHs were enrolled, the estimated outpatient pertussis cases in SHs were calculated by multiplying the number of registered outpatient suspected pertussis cases in SHs by the positive rate of enrolled outpatient suspected cases in SHs.

(4) $$Eip=\frac{Lab-confirmed\;inpatient\;pertussis\;cases\;}{Hpc}$$

*Eip*: estimated number of inpatient pertussis cases in all hospitals of Yiwu; *Hpc*: hospitalization preference coverage rate.

(5) $$Peop=\frac{Lab-confirmed\;outpatient\;pertussis\;cases\;}{Enrolled\;outpatient\;suspected\;pertussis\;cases}$$

*Peop*: positive rate of enrolled outpatient suspected pertussis cases.

(6) $$Eop=\frac{Registered\;outpatient\;suspected\;pertussis\;cases\times Peop}{Mpc}$$

*Eop*: estimated number of outpatient pertussis cases in all hospitals of Yiwu; *Peop*: positive rate of enrolled outpatient suspected pertussis cases.

*Mpc*: medical consultation preference coverage rate.

### 2.5. Data Analysis

Descriptive statistical analyses were performed using frequency distributions and rate calculations. Data analysis was performed using R 3.1.0 software. The estimated pertussis cases in the community, the incidence rate of pertussis in the community, and the percentage of hospitalization for pertussis infection were calculated using Formulas (7)–(9), respectively. The 95% credible intervals (CrIs) of estimated pertussis incidence were calculated using the bootstrap method with 1000 replications, considering the samplings when calculating hospitalization preference coverage rate of the surveillance hospitals (Tables S1 and S2), positive rate of enrolled outpatients with suspected pertussis (Tables S3 and S4), medical consultation preference coverage rate of the surveillance hospitals (Tables S1 and S2), and the medical consultation rate of survey-identified suspected pertussis cases (Tables S1 and S2). The estimated number of pertussis cases in the community (*Epc*), the incidence rate of pertussis in the community (*Ip*), and the percentage of hospitalization for pertussis infection (*Hp*) by age group were also computed using Formulas (7)–(9), with relevant parameters derived from each age group (Tables S1–S8).

(7) $$Epc=\frac{Eip+Eop\;}{Mcs}$$

*Epc*: the estimated number of pertussis cases in the community of Yiwu; *Eip*: estimated number of inpatient pertussis cases in all hospitals of Yiwu.

*Eop*: estimated number of outpatient pertussis cases in all hospitals of Yiwu; *Mcs*, medical consultation rate of survey-identified suspected pertussis cases.

(8) $$Ip=\frac{Epc}{Resident\;population}$$

*Ip*: pertussis incidence per 100,000 person-years; *Epc*: estimated number of pertussis cases in the community of Yiwu.

(9) $$Hp=\frac{Eip}{Epc}$$

*Hp*: percentage hospitalized among those with pertussis; *Eip*: estimated number of inpatient pertussis cases in all hospitals of Yiwu.

*Epc*: estimated number of pertussis cases in the community of Yiwu.

## 3. Results

### 3.1. Medical Consultation Preference and Hospitalization Preference Coverage Rates of Surveillance Hospitals and the Medical Consultation Rate of Survey-Identified Suspected Pertussis Cases

According to the HUAS, a total of 9475 participants were surveyed, among whom 5967 expressed a preference to seek medical consultation at a surveillance hospital and 7529 expressed a preference to be hospitalized at a surveillance hospital, which corresponded to a collective medical consultation coverage preference rate of 63.0% (95% CrI: 62.0–64.0%) and a collective hospitalization coverage preference rate of 79.5% (95% CrI: 78.7–80.3%). The medical consultation preference and hospitalization preference coverage rates are summarized by age group in Tables S1 and S2.

Among the 9475 HUAS participants surveyed, 252 suspected pertussis cases (named survey-identified suspected pertussis cases) were identified based on the cough duration and typical pertussis-like symptoms that the survey participants presented in the past month. Among the 252 survey-identified suspected pertussis cases, 189 visited hospitals when they had a cough, corresponding to a medical consultation rate of 75.0% (95% CrI: 69.4–80.2). The medical consultation rates of survey-identified suspected pertussis cases by age group are summarized in Table S1. Since only 15 survey-identified suspected pertussis cases were under 1 year of age, the medical consultation rate for infants under 1 year of age was used as a reference for the medical consultation rate of infants at different months of age, which was 100.0%.

### 3.2. General Information on Laboratory-Confirmed Pertussis Cases

During the study period of 1 June 2021 to 31 May 2022, a total of 873 patients with suspected pertussis were enrolled, of whom 184 patients (21.1%) were identified as having lab-confirmed pertussis (Table 1). Among them, 106 (57.6%) participants were male, and 37 (20.1%) were inpatients. Laboratory-confirmed pertussis cases were predominantly from three age groups: 6–19 years (26.1%), <1 year (24.5%), and 4–5 years (21.7%), which collectively accounted for 72.3% of the total cases. The median cough duration before diagnosis among participants with lab-confirmed pertussis was 17.0 days (interquartile range [IQR] 14.0–25.8). The most common symptoms among the participants with lab-confirmed pertussis were paroxysmal cough (50.5%), post-tussive emesis (42.4%), worsening cough at night (41.3%), expectoration (33.7%), inspiratory whooping (12.5%), and runny nose (11.4%).

### 3.3. Estimated Community Pertussis Incidence Rate by Age Group

Among all ages, the estimated community incidence rate of pertussis in Yiwu during the 12-month study period was 108.3 (95% CrI: 91.7–126.4) per 100,000. The pertussis incidence rate peaked in the 4–5 year age group (with 1154.3 [95% CrI: 817.4–1553.5] per 100,000), before declining with increasing age thereafter (Table 2 and Figure S2). Notably, infants aged 1 year (836.4 [95% CrI: 434.0–1308.8] per 100,000) and <1 year (832.7 [95% CrI: 614.9–1059.4] per 100,000) also had high pertussis incidence rate. The pertussis incidence rate in the 2–3 year age group (629.6 [95% CrI: 385.7–925.7] per 100,000) was almost half that observed in the 4–5 year age group.

A more detailed breakdown of the pertussis incidence rate in infants younger than 1 year by ages in months is summarized in Table 3 and Figure S3. Infants aged 4 months, 5 months, and 3 months had the highest pertussis incidence rates estimated at 1209.5 [95% CrI: 841.5–1636.5] per 100,000; 1035.4 [95% CrI: 359.2–1729.4] per 100,000; and 816.7 [95% CrI: 571.5–1081.4] per 100,000, respectively. Infants aged 6–11 months (607.7 [95% CrI: 125.1–1102.5] per 100,000) and <3 months (544.8 [95% CrI: 434.9–690.0] per 100,000) had comparably lower pertussis incidence rates than the other groups aged < 1 year.

### 3.4. Percentage Hospitalized Among Those with Pertussis

The overall percentage hospitalized among those with pertussis across all ages was 2.3% (95% CrI: 2.0–2.7) (Table 4 and Figure S4). The percentage hospitalized for pertussis infections was highest in the youngest age (infants aged < 3 months) group at 63.1% (95% CrI: 51.5–86.1). Hospitalization rates remained high in infants aged 3 months (61.4%; 95% CrI: 47.7–83.0), and gradually decreased through ages 3–5 months. Hospitalization rates were generally very low from age 6–11 months and remained low throughout childhood, adolescence, and adulthood, with a secondary peak in the elderly aged ≥ 60 years (6.7%; 95% CrI 2.2–91.7).

## 4. Discussion

In this study, we conducted active pertussis surveillance in Yiwu, Zhejiang province, China, and estimated the incidence rate of pertussis in the community and hospitalization rates of pertussis cases during a 12-month period. The overall estimated incidence was 108.3 per 100,000 person-years, with children aged 4–5 years experiencing the highest incidence at 1154.3 per 100,000 person-years. Notably, infants younger than 3 months had the highest hospitalization rate, with an estimated 63.0% of pertussis cases requiring hospitalization. Although the incidence was low in elderly aged ≥ 60 years, the hospitalization rate was rather high (6.7%). These findings highlight the age-specific burden of pertussis, particularly among young children and infants, who remain the most vulnerable to severe disease. The study also emphasizes the importance of timely vaccination to prevent those too young to be fully immunized and elderly aged ≥ 60 years from contracting severe pertussis.

The total estimated pertussis incidence rate in this study (108.3 per 100,000) was approximately 33 times higher than the total incidence of pertussis reported in Zhejiang during the same period, as determined by passive surveillance (3.24 per 100,000). The total incidence of pertussis in Zhejiang was estimated by calculating the number of reported pertussis cases in the Zhejiang province [22] and divided by the average long-term residents data of the Zhejiang province from 2021 to 2022 [23]. Moreover, this underestimation in previous reports compared with the current study appears to be across almost every age group [14,24,25,26]. These findings indicate that there is an urgent need to strengthen pertussis surveillance across all age groups in China to determine the true pertussis burden across the country.

We observed that children aged 4–5 years had the highest pertussis incidence rate, while the passive surveillance of pertussis in China and other countries in recent years indicated that infants aged less than one year had the highest pertussis incidence rate [14,24,25,26]. This difference implied that the disease burden of children aged 4–5 years might be highly underestimated in passive surveillance. A high pertussis incidence rate among older children may lead to clustered outbreaks in kindergartens and primary schools. In fact, among fifteen public health emergencies of pertussis outbreaks reported in China in 2022, two occurred in kindergartens, eight in primary schools, and the remaining five in other institutions/workplaces [14]. It is widely recognized that waning immunity could contribute to an increased incidence in preschool children [10,14,27]. Indeed, among the 194 WHO member states, 68 (35.1%) recommend a 5-dose schedule, which includes a preschool booster for children [28]. However, except for a series of primary vaccinations at 3, 4, and 5 months of age, followed by a booster at 18 months of age, there are no pre-school boosters scheduled for children in mainland China. Our study highlights that while the incidence of pertussis is notably high among infants aged 3 to 5 months, there is a significant decrease in the incidence after 5 months, suggesting that the acellular pertussis vaccine (DTaP) provides good short-term protection. However, the marked increase in incidence at ages 4 to 5 years indicates a waning of immunity earlier than previously assumed. This finding is consistent with a prospective study conducted in Japan, which monitored over 1000 children aged 0 to 7 years over a 10-year period. This study reported a decline in the protective effectiveness of the acellular pertussis vaccine from an initial 100% post-vaccination to approximately 50% by ages 4 to 5 [29]. Given the evidence of early waning immunity, there may be a need to consider a booster to enhance protection prior to the significant increase in incidence at ages 4 to 5.

Our study also revealed important insights into the vulnerability of infants aged under 1 year and elders aged ≥ 60 years. When analyzing the incidence rates in infants by age in months, we found that those aged 3–5 months had the highest incidence of pertussis, significantly surpassing the incidence rate among the same age groups observed in passive surveillance of pertussis in China between 2018 and 2022 [14,24]. This suggests that the true burden of pertussis in these vulnerable age groups may also be underestimated in passive surveillance, highlighting the importance of active surveillance for more accurate detection and the need for targeted vaccination strategies or earlier interventions to protect infants during this critical period. Moreover, our study showed that infants aged < 6 months had the highest percentage of hospitalization for pertussis compared to other age groups. The percentage of hospitalization for pertussis also increased in older adults aged ≥ 60 years compared to other age groups (excluding young infants). These findings indicated that infants aged < 6 months and older adults aged ≥ 60 years were more likely to experience severe pertussis, underscoring the need for enhanced protective measures, such as timely vaccination and early diagnosis, to reduce the risk of severe outcomes in these high-risk groups.

Although our study offered valuable insights into the epidemiology of pertussis, several limitations need to be acknowledged. First, according to our case enrollment criteria, nearly all the enrolled suspected pertussis cases exhibited typical pertussis-like symptoms, which may have led to the omission of atypical cases. Second, this study was conducted during the COVID-19 pandemic, and public health measures such as wearing masks, physical distancing, and isolation may have also reduced the incidence of respiratory infectious diseases and the medical seeking behaviors of residents in the participating community [7]. On one hand, patients may have been less likely to visit the clinic because of concerns about cross-contamination; on the other hand, patients’ increased attention to respiratory illnesses increased the probability of visiting the clinic. The dual impact of COVID-19 could have affected the data’s representativeness in this population-based active surveillance, but the exact impact on pertussis incidence is challenging to evaluate precisely. Third, although the SHs were selected due to the significant utilization by residents in Yiwu, discrepancies between the preference coverage rate of SHs, as estimated by the HUAS, and the real coverage rate of SHs could lead to inaccuracies. Fourth, the number of pertussis cases is likely to be underestimated due to the dependence on PCR and culture, which have a restricted detection window and reduced sensitivity as the disease advances [21]. Pertussis cases may have been missed if samples were collected beyond the optimal period for laboratory confirmation using PCR and culture. Future research addressing these limitations will be essential to further our knowledge of pertussis epidemiology and provide more comprehensive insights to better inform public health interventions and vaccination strategies.

## 5. Conclusions

This active population-based surveillance suggests that the incidence of pertussis in Yiwu, China, has generally been underestimated and that there is a high estimated incidence of the disease in the community across all age groups. The highest estimated incidence of pertussis occurs among young children aged 4–5 years and those aged 1 year, while those too young (aged ≤ 4 months) to be fully vaccinated against the disease had the highest hospitalization rates. Large-scale active surveillance of pertussis is urgently needed to fully understand pertussis epidemiology and inform policy decisions on pertussis control.

## Figures and Tables

**Table 1:** Characteristics of those with laboratory-confirmed pertussis in Yiwu, Zhejiang province, from 1 June 2021 to 31 May 2022 *.

Characteristics	Laboratory-Confirmed Pertussis Cases, n (%)
Participants	184
Gender	
Male	106 (57.6)
Female	78 (42.4)
Type	
Inpatient	37 (20.1)
Outpatient	147 (79.9)
Age, median (IQR), years	4.70 (1.1–7.5)
Age group	
<1 y	45 (24.5)
1 y	10 (5.4)
2–3 y	20 (10.9)
4–5 y	40 (21.7)
6–19 y	48 (26.1)
20–59 y	19 (10.3)
≥60 y	2 (1.1)
Cough duration, median (IQR), days	17.0 (14.0–25.8)
Clinical manifestations	
Paroxysmal cough	93 (50.5)
Post-tussive emesis	78 (42.4)
Inspiratory whooping	23 (12.5)
Worsening cough at night	76 (41.3)
Apnea	5 (2.7)
Expectoration	62 (33.7)
Polypnea	5 (2.7)
Cyanosis	3 (1.6)
Fever	3(1.6)
Runny nose	21 (11.4)
Sore throat	10 (5.4)
Hoarseness	10 (5.4)
Weakness	0 (0.0)
Lacrimation	5 (2.7)
Headache	1 (0.5)
^a^ Other symptoms	5 (2.7)

* Values are n (%) unless otherwise indicated. IQR, interquartile range; n, number of participants; y, year. ^a^ ‘Other symptoms’ included eclampsia, hemoptysis, chills, ague, chest pain, night sweats, myalgia, and arthralgia.

**Table 2:** Pertussis incidence by age group in Yiwu, Zhejiang province, from 1 June 2021 to 31 May 2022.

Age Group	*Eip*, n	*Eop*, n	*Mcs*, %	*Epc*, n	Resident Population, N	*Ip* (95% CrI ^a^)
<1 y	29.6	84.8	100.0	114.4	13,738	832.7 (614.9–1059.4)
1 y	0.0	149.5	90.0	166.2	19,872	836.4 (434.0–1308.8)
2–3 y	3.5	187.7	82.7	231.2	36,722	629.6 (385.7–925.7)
4–5 y	5.9	353.3	86.4	415.7	36,013	1154.3 (817.4–1553.5)
6–19 y	3.7	492.1	45.0	1101.9	220,586	499.5 (331.2–828.6)
20–59 y	0.0	94.5	62.8	150.5	1,341,805	11.2 (6.3–17.7)
≥60 y	1.4	14.0	73.3	21.0	190,654	11.0 (0.8–32.6)
All groups	46.5	1463.4	75.0	2013.2	1,859,390	108.3 (91.7–126.4)

^a^ Calculated by the bootstrap method, the calculation involved the 95% CrI of hospitalization coverage preference rates of SHs, the 95% CrI of the positive rate of enrolled outpatient suspected pertussis cases, the 95% CrI of the medical consultation coverage preference rates of SHs, and the 95% CrI of the medical consultation rates of survey-identified suspected pertussis cases (all shown in the online resources). *Eip*, estimated inpatient pertussis cases in all hospitals of Yiwu, calculated with Formula (4) in Section 2.4; *Eop*, estimated outpatient pertussis cases in all hospitals of Yiwu, calculated with Formulas (5) and (6) in Section 2.4; *Mcs*, medical consultation rate of survey-identified suspected pertussis cases, calculated with Formula (3) in Section 2.2; *Epc*, estimated pertussis cases in community of Yiwu, calculated with Formula (7) in Section 2.5; *Ip*, pertussis incidence per 100,000 person-years, calculated with Formula (8) in Section 2.5; CrI, confidence interval; n, the number of *Eip*/*Eop*/*Epc*; N, the number of resident population; y, year.

**Table 3:** Pertussis incidence among infants aged younger than 1 year, stratified by month of age, in Yiwu, Zhejiang province, from 1 June 2021 to 31 May 2022.

Age Group	*Eip*, n	*Eop*, n	*Mcs*, *%*	*Epc*, n	Resident Population, N	*Ip* (95% CrI ^a^)
<3 m	13.2	7.7	100.0	20.9	3836	544.8 (434.9–690.0)
3 m	5.8	3.7	100.0	9.4	1151	816.7 (571.5–1081.4)
4 m	7.3	6.6	100.0	13.8	1141	1209.5 (841.5–1636.5)
5 m	3.6	8.3	100.0	12.0	1159	1035.4 (359.2–1729.4)
6–11 m	0.0	39.2	100.0	39.2	6451	607.7 (125.1–1102.5)
All groups	29.6	84.8	100.0	114.4	13,738	832.7 (614.9–1059.4)

^a^ Calculated by the bootstrap method, the calculation involved the 95% CrI of hospitalization coverage preference rates of SHs, the 95% CrI of the positive rate of enrolled outpatient suspected pertussis cases, the 95% CrI of the medical consultation coverage preference rates of SHs, and the 95% CrI of the medical consultation rates of survey-identified suspected pertussis cases (all shown in the online resources). *Eip*, estimated inpatient pertussis cases in all hospitals of Yiwu, calculated with Formula (4) in Section 2.4; *Eop*, estimated outpatient pertussis cases in all hospitals of Yiwu, calculated with Formulas (5) and (6) in Section 2.4; *Mcs*, medical consultation rate of survey-identified suspected pertussis cases; since only 15 survey-identified suspected pertussis cases were under 1 year of age, the medical consultation rate for infants under 1 year of age (100.0%) was used as a reference for the medical consultation rate of infants at different months of age; *Epc*, estimated pertussis cases in community of Yiwu, calculated with Formula (7) in Section 2.5; *Ip*, pertussis incidence per 100,000 person-years, calculated with Formula (8) in Section 2.5; CrI, confidence interval; n, the number of *Eip*/*Eop*/*Epc*; N, the number of resident population; y, year.

**Table 4:** Percentage hospitalized among those with pertussis in Yiwu, Zhejiang province, from 1 June 2021 to 31 May 2022.

Age Group	*Eip*, n	*Epc*, n	Percentage Hospitalized Among Those with Pertussis ^a^, %	95% CrI ^b^, %
<3 m	13.2	20.9	63.1	51.5–86.1
3 m	5.8	9.4	61.4	47.7–83.0
4 m	7.3	13.8	52.6	40.0–73.6
5 m	3.6	12.0	30.3	18.2–58.0
6–11 m	0.0	39.2	0.0	0.0–0.0
1 y	0.0	166.2	0.0	0.0–0.0
2–3 y	3.5	231.2	1.5	1.0–2.5
4–5 y	5.9	415.7	1.4	1.0–2.0
6–19 y	3.7	1101.9	0.3	0.2–0.5
20–59 y	0.0	150.5	0.0	0.0–0.0
≥60 y	1.4	21.0	6.7	2.2–91.7
All groups	46.5	2013.2	2.3	2.0–2.7

^a^ Calculated with Formula (9) in Section 2.5; ^b^ Calculated by the bootstrap method. *Eip*, estimated inpatient pertussis cases in all hospitals of Yiwu, calculated with Formula (4) in Section 2.4; *Epc*, estimated pertussis cases in the community of Yiwu, calculated with Formula (7) in Section 2.5; CrI, confidence interval; m, month; y, year.

## Data Availability

The datasets used and/or analyzed during the current study are available from the corresponding author upon reasonable request.

## Supplementary Materials

The following supporting information can be downloaded at: https://www.mdpi.com/article/10.3390/microorganisms12112186/s1, Figure S1: Flow chart regarding the estimation of pertussis incidence and percentage of the population hospitalized for pertussis in the community; Figure S2. Incidence rates (with 95%CrI) by age groups in Yiwu, Zhejiang province from 1 June 2021 to 31 May 2022; Figure S3. Incidence rates (with 95%CrI) among infants aged younger than 1 year, stratified by month of age, in Yiwu, Zhejiang province from 1 June 2021 to 31 May 2022, Zhejiang province from 1 June 2021 to 31 May 2022; Figure S4. Percentage hospitalized among those with pertussis (with 95%CrI) in Yiwu, Zhejiang province, from 1 June 2021 to 31 May 2022; Table S1: Medical consultation preference and hospitalization preference coverage rates and medical consultation rates of survey-identified suspected pertussis cases among all age groups in Yiwu, Zhejiang province, from 2019 to 2020; Table S2. Medical consultation preference and hospitalization preference coverage rates by age group among infants younger than 1 year stratified by month of age in Yiwu, Zhejiang province, from 2019 to 2020; Table S3. Pertussis positive rate of enrolled outpatient suspected pertussis cases in all age groups of Yiwu, Zhejiang province, from 1 June 2021 to 31 May 2022; Table S4. Pertussis positive rate of enrolled outpatient suspected pertussis outpatient cases in infants younger than 1 year, stratified by month of age, in Yiwu, Zhejiang province, from 1 June 2021 to 31 May 2022; Table S5. Estimated outpatient pertussis cases among all age groups in all hospitals of Yiwu, Zhejiang province, from 1 June 2021 to 31 May 2022; Table S6. Estimated outpatient pertussis cases among infants younger than 1 year, stratified by month of age, in in all hospitals of Yiwu, Zhejiang province, from 1 June 2021 to 31 May 2022; Table S7. Estimated inpatient pertussis cases among all age groups in all hospitals of Yiwu, Zhejiang province, from 1 June 2021 to 31 May 2022; Table S8. Estimated inpatient pertussis cases among infants younger than 1 year, stratified by month of age, in all hospitals of Yiwu, Zhejiang province, from 1 June 2021 to 31 May 2022.
