# Supplementary material for: Underestimated Incidence Rate of Pertussis in the Community: Results from Active Population-Based Surveillance in Yiwu, China
Source: Microorganisms. 2024 Oct 30;12(11):2186. doi: 10.3390/microorganisms12112186 (PMC11596255; doi:10.3390/microorganisms12112186)
Supplement: Supplementary file 1 [file microorganisms-12-02186-s001.zip › microorganisms-3247286-supplementary.pdf]

**Online Resources**

# Underestimated Incidence Rate of Pertussis in the Community: Results from Active Population-Based Surveillance in Yiwu, China

Hanying Dai[1,†], Hanqing He[2,†], Juan Xu[1], Yao Zhu[2], Tao Fu[3], Bohan Chen[1], Jie Li[1], Yuan Gao[1], Aiping Qin[1], Maojun Zhang[1], Zhujun Shao[1]

[†]These authors contributed equally

1. *National Key Laboratory of Intelligent Tracking and Forecasting for Infectious Diseases, National Institute for Communicable Disease Control and Prevention, Chinese Center for Disease Control and Prevention, Beijing, 102206, China.*
2. *Zhejiang Provincial Center for Disease Control and Prevention, Hangzhou, China.*
3. *Yiwu District Center for Disease Control and Prevention, Jinhua, China.*

**Corresponding author**: Zhujun Shao (shaozhujun@icdc.cn)

**Address:** National Key Laboratory of Intelligent Tracking and Forecasting for Infectious Diseases, National Institute for Communicable Disease Control and Prevention, Chinese Center for Disease Control and Prevention, Beijing, 102206, China.

**Online Resource Table S1.** Medical consultation preference and hospitalization preference coverage rates and medical consultation rates of survey-identified suspected pertussis cases among all age groups in Yiwu, Zhejiang province, from 2019 to 2020.

| Age group | Participants, N | Preference to seek medical consultation at SHs, n | *Mpc*[a], % (95% CrI[d]) | Preference to be hospitalized at SHs, n | *Hpc*[b], % (95% CrI[d]) | Survey-identified suspected pertussis cases, n | Medical consulted suspected pertussis cases, n | *Mcs*[c], % (95% CrI[d]) |
|---|---|---|---|---|---|---|---|---|
| <1y | 756 | 578 | 76.5 (73.3–79.4) | 638 | 84.4 (81.8–87.0) | 15 | 15 | 100.0 (100.0–100.0) |
| 1y | 623 | 434 | 69.7 (66.1–73.2) | 514 | 82.5 (79.5–85.2) | 20 | 18 | 90.0 (75.0–100.0) |
| 2–3y | 1171 | 871 | 74.4 (71.7–76.9) | 998 | 85.2 (83.3–87.2) | 75 | 62 | 82.7 (73.6–91.4) |
| 4–5y | 592 | 443 | 74.8 (71.1–78.2) | 502 | 84.8 (81.8–87.5) | 44 | 38 | 86.4 (75.0–95.2) |
| 6–19y | 894 | 628 | 70.3 (67.5–73.3) | 721 | 80.7 (78.1–83.1) | 40 | 18 | 45.0 (29.4–62.2) |
| 20–59y | 4541 | 2581 | 56.8 (55.4–58.3) | 3,514 | 77.4 (76.2–78.6) | 43 | 27 | 62.8 (47.6–77.8) |
| ⩾60y | 898 | 432 | 48.1 (44.7–51.3) | 642 | 71.5 (68.4–74.5) | 15 | 11 | 73.3 (50.0–93.8) |
| All groups | 9475 | 5967 | 63.0 (62.0–64.0) | 7,529 | 79.5 (78.7–80.3) | 252 | 189 | 75.0 (69.4–80.2) |

[a] *Mpc*, medical consultation preference coverage rate, calculated using formula (2) in section 2.2 ; [b] *Hpc*, hospitalization preference coverage rate, calculated using formula (1) in section 2.2; [c] *Mcs*, medical consultation rate of survey-identified suspected pertussis cases, calculated using formula (3) in section 2.2 ; [d] Calculated by bootstrap methods. CrI; credible intervals; N, the total number of participants; n, the total number of participants fulfilling the criteria listed; SHs, surveillance hospitals; y, year.

**Online Resource Table S2.** Medical consultation preference and hospitalization preference coverage rates by age group among infants younger than 1 year stratified by month of age in Yiwu, Zhejiang province, from 2019 to 2020.

| Age group | Participants, N | Preference to seek medical consultation at SHs, n | *Mpc*[a], % (95% CrI[c]) | Preference to be hospitalized at SHs, n | *Hpc*[b], % (95% CrI[c]) |
|---|---|---|---|---|---|
| <3m | 157 | 122 | 77.7 (71.3–84.1) | 131 | 83.4 (77.7–89.2) |
| 3m | 67 | 55 | 82.1 (71.6–91.0) | 58 | 86.6 (77.6–94.0) |
| 4m | 75 | 57 | 76.0 (65.3–85.3) | 62 | 82.7 (73.3–90.7) |
| 5m | 68 | 49 | 72.1 (60.3–82.4) | 56 | 82.4 (73.5–91.2) |
| 6–11m | 389 | 295 | 75.8 (71.5–80.0) | 331 | 85.1 (81.5–88.7) |
| All groups | 756 | 578 | 76.5 (73.3–79.4) | 638 | 84.4 (81.8–87.0) |

[a] *Mpc*, medical consultation preference coverage rate, calculated using formula (2) in section 2.2 ; [b] *Hpc*, hospitalization preference coverage rate, calculated using formula (1) in section 2.2; [c] Calculated by bootstrap methods. CrI; credible intervals; N, the total number of participants; n, the total number of participants fulfilling the criteria listed; SHs, surveillance hospitals; m, month.

**Online Resource Table S3.** Pertussis positive rate of enrolled outpatient suspected pertussis cases in all age groups of Yiwu, Zhejiang province, from June 1, 2021, to May 31, 2022.

| Age Group | Enrolled outpatient suspected pertussis cases, N | Lab-confirmed outpatient pertussis cases, n | *Peop*[a], % (95% CrI[b]) |
|---|---|---|---|
| <1y | 53 | 20 | 37.7 (24.5–50.9) |
| 1y | 30 | 10 | 33.3 (16.7–50.0) |
| 2–3y | 109 | 17 | 15.6 (9.2–22.9) |
| 4–5y | 151 | 35 | 23.2 (17.2–30.5) |
| 6–19y | 151 | 45 | 29.8 (22.5–37.1) |
| 20–59y | 173 | 19 | 11.0 (6.4–15.6) |
| ≥60y | 18 | 1 | 5.6 (0.0–16.7) |
| All groups | 685 | 147 | 21.5 (18.4–24.5) |

[a] *Peop*, positive rate of enrolled outpatient suspected pertussis cases, calculated using formula (5) in section 2.4; [b] Calculated by the bootstrap method; CrI, credible interval; N, the total number of enrolled suspected pertussis outpatient cases; n, the total number of participants fulfilling the criteria listed; y, year

**Online Resource Table S4.** Pertussis positive rate of enrolled outpatient suspected pertussis outpatient cases in infants younger than 1 year, stratified by month of age, in Yiwu, Zhejiang province, from June 1, 2021, to May 31, 2022.

| Age group | Enrolled outpatient suspected pertussis cases, N | Lab-confirmed outpatient pertussis cases, n | *Peop*[a], % (95% CrI[b]) |
|---|---|---|---|
| <3m | 7 | 4 | 54.7 (28.6–85.7) |
| 3m | 6 | 3 | 50.0 (16.7–83.3) |
| 4m | 11 | 5 | 45.5 (18.2–72.7) |
| 5m | 8 | 3 | 37.5 (0.0–75.0) |
| 6–11m | 21 | 5 | 23.8 (4.8–42.9) |
| All groups | 53 | 20 | 37.7 (24.5–50.9) |

[a] *Peop*, positive rate of enrolled outpatient suspected pertussis cases, calculated using formula (5) in section 2.4; [b] Calculated by the bootstrap method; CrI, credible interval; N, the total number of enrolled suspected pertussis outpatient cases; n, the total number of participants fulfilling the criteria listed; m, month.

**Online Resource Table S5.** Estimated outpatient pertussis cases among all age groups in all hospitals of Yiwu, Zhejiang province, from June 1, 2021, to May 31, 2022.

| Age group | Registered outpatient suspected pertussis cases, n | *Peop*[a], % | *Mpc*[b], % | *Eop*[c], N |
|---|---|---|---|---|
| <1y | 172 | 37.7 | 76.5 | 84.8 |
| 1y | 313 | 33.3 | 69.7 | 149.5 |
| 2–3y | 895 | 15.6 | 74.4 | 187.7 |
| 4–5y | 1,139 | 23.2 | 74.8 | 353.3 |
| 6–19y | 1,161 | 29.8 | 70.3 | 492.1 |
| 20–59y | 488 | 11.0 | 56.8 | 94.5 |
| ≥60y | 120 | 5.6 | 48.1 | 14.0 |
| All groups | 4288 | 21.5 | 63 | 1463.4 |

[a] *Peop*, positive rate of enrolled outpatient suspected pertussis cases, calculated using formula (5) in section 2.4; [b]*Mpc*, medical consultation preference coverage rate of SHs, calculated using formula (2) in section 2.2; [c]*Eop*, estimated outpatient pertussis cases in all hospitals of Yiwu,calculated using formula (6) in section 2.4 ; y, year; n, the total number of registered outpatient suspected pertussis cases in SHs; N, the total number of estimated outpatient pertussis cases in all hospitals of Yiwu.

**Online Resource Table S6.** Estimated outpatient pertussis cases among infants younger than 1 year, stratified by month of age, in in all hospitals of Yiwu, Zhejiang province, from June 1, 2021, to May 31, 2022.

| Age group | Registered outpatient suspected pertussis cases, n | *Peop*[a], % | *Mpc*[b], % | *Eop*[c], N |
|---|---|---|---|---|
| <3m | 11 | 54.7 | 77.7 | 7.7 |
| 3m | 6 | 50.0 | 82.1 | 3.7 |
| 4m | 11 | 45.5 | 76 | 6.6 |
| 5m | 16 | 37.5 | 72.1 | 8.3 |
| 6–11m | 125 | 23.8 | 75.8 | 39.2 |
| All groups | 172 | 37.7 | 76.5 | 84.8 |

[a] *Peop*, positive rate of enrolled outpatient suspected pertussis cases, calculated using formula (5) in section 2.4; [b]*Mpc*, medical consultation preference coverage rate of SHs, calculated using formula (2) in section 2.2; [c]*Eop*, estimated outpatient pertussis cases in all hospitals of Yiwu,calculated using formula (6) in section 2.4 ; m, month; n, the total number of registered outpatient suspected pertussis cases in SHs; N, the estimated total number of estimated outpatient pertussis cases in all hospitals of Yiwu.

**Online Resource Table S7.** Estimated inpatient pertussis cases among all age groups in all hospitals of Yiwu, Zhejiang province, from June 1, 2021, to May 31, 2022.

| Age Group | Lab-confirmed intpatient pertussis cases, n | *Hpc*[a], % | *Eip*[b], N |
|---|---|---|---|
| <1y | 25 | 84.4 | 29.6 |
| 1y | 0 | 82.5 | 0.0 |
| 2–3y | 3 | 85.2 | 3.5 |
| 4–5y | 5 | 84.8 | 5.9 |
| 6–19y | 3 | 80.7 | 3.7 |
| 20–59y | 0 | 77.4 | 0.0 |
| ≥60y | 1 | 71.5 | 1.4 |
| All groups | 37 | 79.5 | 46.5 |

[a] *Hpc*, hospitalization preference coverage rate of SHs, calculated using formula (1) in section 2.2; [b]*Eip*, estimated inpatient pertussis cases in all hospitals of Yiwu; y, year; n, the total number of lab-confirmed inpatient pertussis cases in SHs; N, the total number of estimated inpatient pertussis cases in all hospitals of Yiwu.

**Online Resource Table S8.** Estimated inpatient pertussis cases among infants younger than 1 year, stratified by month of age, in all hospitals of Yiwu, Zhejiang province, from June 1, 2021, to May 31, 2022.

| Age Group | Lab-confirmed intpatient pertussis cases, n | *Hpc*[a] , % | *Eip*[b], N |
|---|---|---|---|
| <3m | 11 | 83.4 | 13.2 |
| 3m | 5 | 86.6 | 5.8 |
| 4m | 6 | 82.7 | 7.3 |
| 5m | 3 | 82.4 | 3.6 |
| 6–11m | 0 | 85.1 | 0.0 |
| All groups | 25 | 84.4 | 29.6 |

[a] *Hpc*, hospitalization preference coverage rate of SHs, calculated using formula (1) in section 2.2; [b]*Eip*, estimated inpatient pertussis cases in all hospitals of Yiwu; m, month; n, the total number of lab-confirmed inpatient pertussis cases in SHs; N, the total number of estimated inpatient pertussis cases in all hospitals of Yiwu.

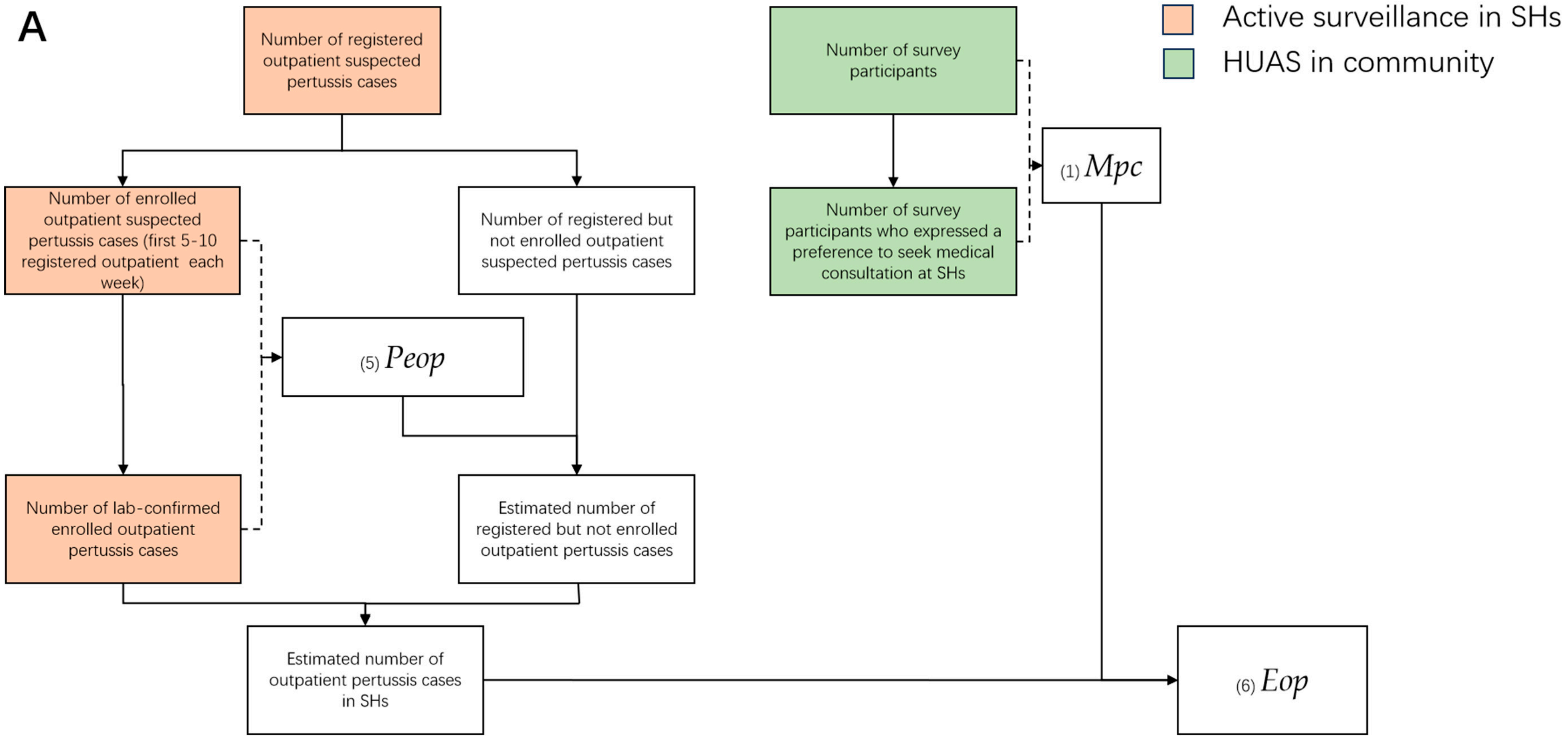
A
Active surveillance in SHs
HUAS in community
Number of registered outpatient suspected pertussis cases
Number of survey participants
(1) Mpc
Number of enrolled outpatient suspected pertussis cases (first 5-10 registered outpatient each week)
Number of registered but not enrolled outpatient suspected pertussis cases
Number of survey participants who expressed a preference to seek medical consultation at SHs
(5) Peop
Number of lab-confirmed enrolled outpatient pertussis cases
Estimated number of registered but not enrolled outpatient pertussis cases
Estimated number of outpatient pertussis cases in SHs
(6) Eop

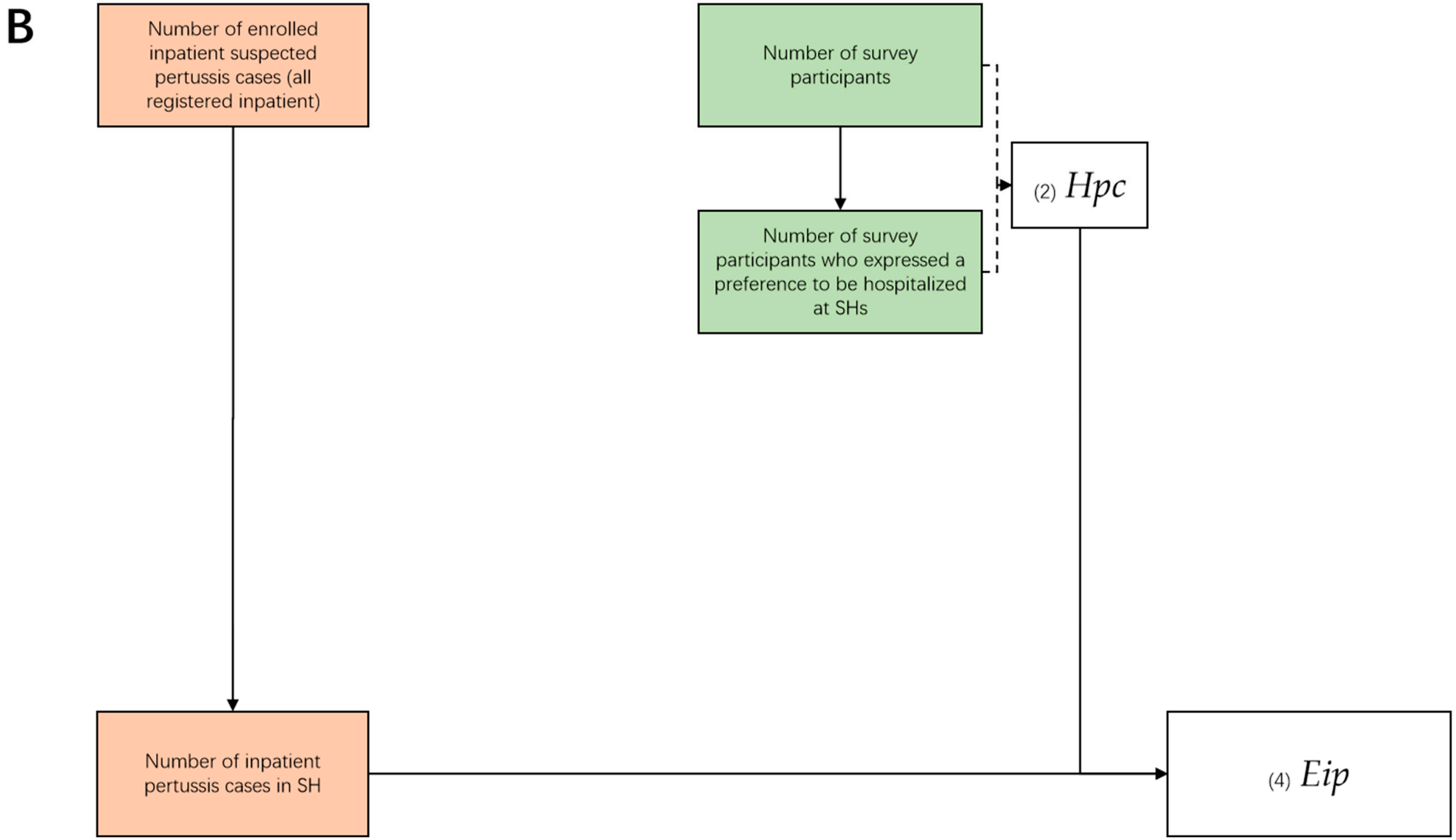
B
Number of enrolled inpatient suspected pertussis cases (all registered inpatient)
Number of survey participants
(2) Hpc
Number of survey participants who expressed a preference to be hospitalized at SHs
Number of inpatient pertussis cases in SH
(4) Eip

C

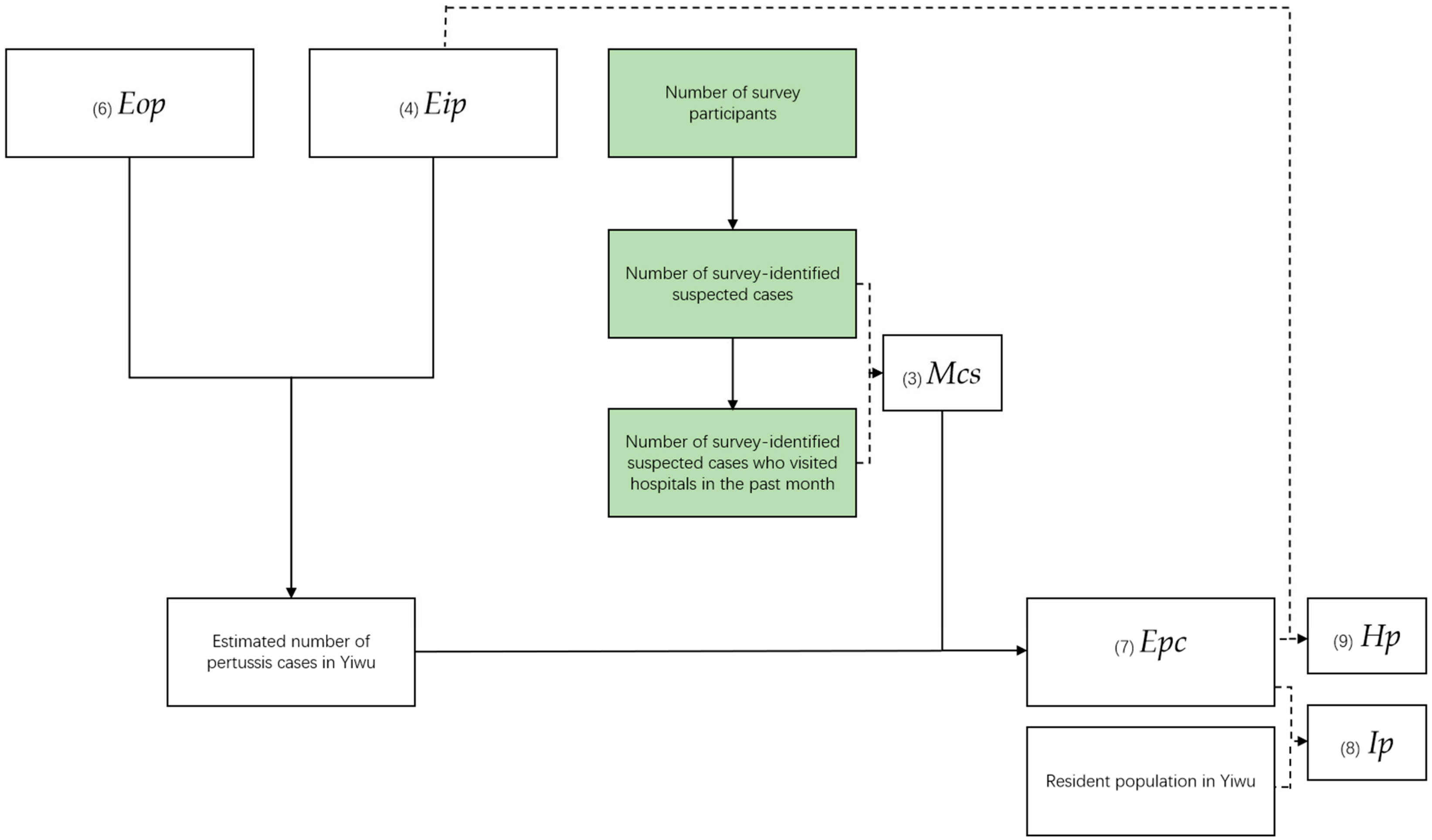


**Online Resource Figure S1.** Flow chart regarding the estimation of pertussis incidence and percentage of the population hospitalized for pertussis in the community. **(A)** Estimated number of outpatient pertussis cases in all hospitals of Yiwu. *Mpc*, medical consultation preference coverage rate

of surveillance hospitals, calculated using formula (1) in section 2.2; *Peop*, positive rate of enrolled outpatient suspected pertussis cases, calculated using formula (5) in section 2.4; *Eop*, estimated outpatient pertussis cases in all hospitals of Yiwu, calculated using formula (6) in section 2.4. **(B)** Estimated number of inpatient pertussis cases in all hospitals of Yiwu. *Hpc*, hospitalization preference coverage rate of SHs, calculated using formula (2) in section 2.2; *Eip*,estimated inpatient pertussis cases in all hospitals of Yiwu, calculated using formula (4) in section 2.4; **(C)** Estimated incidence rate of pertussis and percentage hospitalized among those with pertussis in community. *Epc*, estimated pertussis cases in community of Yiwu, calculated using formula (7) in section 2.5; *Mcs*, medical consultation rate of survey-identified suspected pertussis cases, calculated using formula (3) in section 2.2; *Ip*, pertussis incidence per 100,000 person-years, calculated using formula (8) in section 2.5. *Hp*, percentage hospitalized among those with pertussis, calculated using formula (9) in section 2.5.

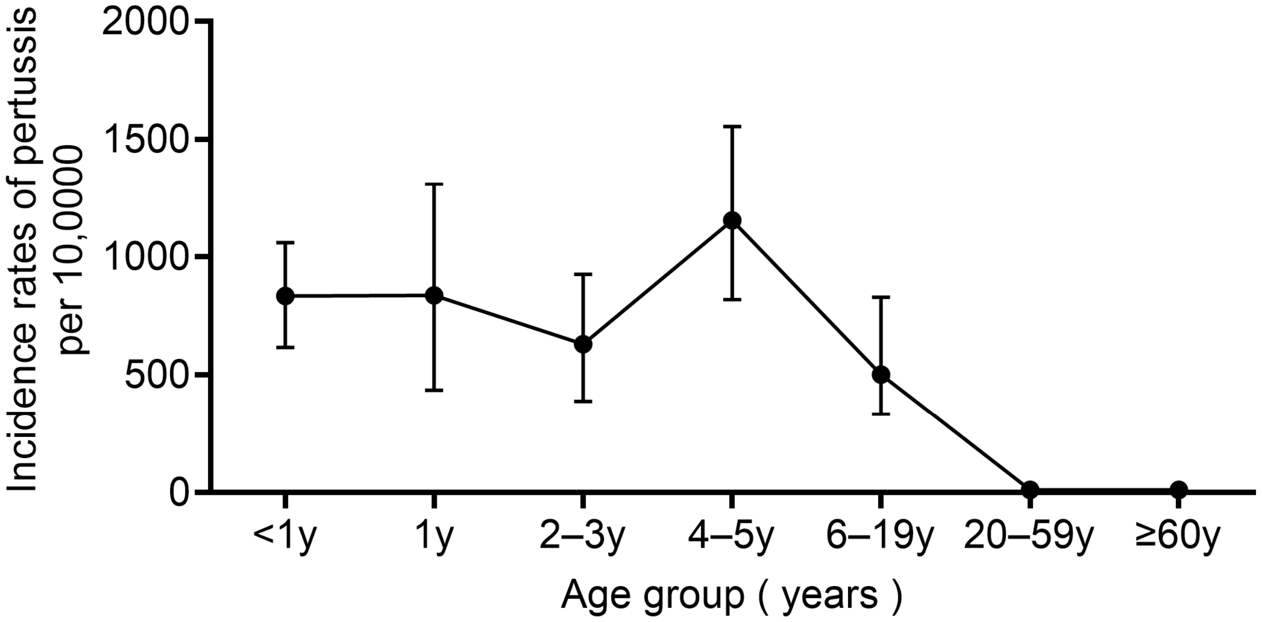


**Online Resource Figure S2.** Incidence rates (with 95%CrI) by age groups in Yiwu, Zhejiang province from June 1, 2021, to May 31, 2022. CrI, credible interval; y, year.

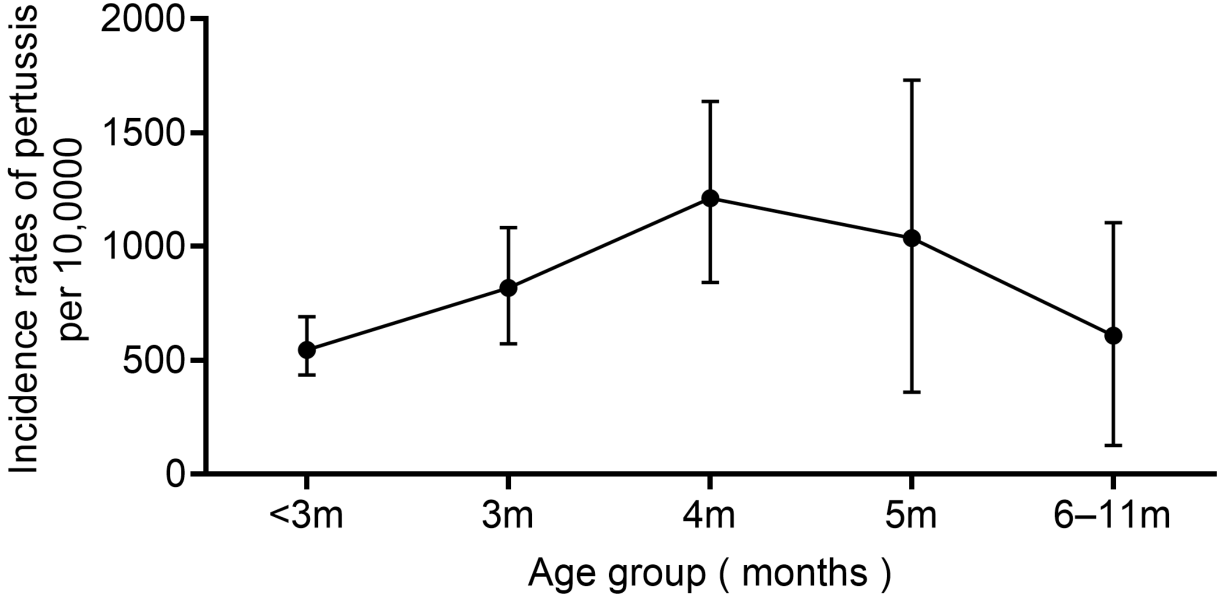


**Online Resource Figure S3.** Incidence rates (with 95%CrI) among infants aged younger than 1 year, stratified by month of age, in Yiwu, Zhejiang province from June 1, 2021, to May 31, 2022., Zhejiang province from June 1, 2021, to May 31, 2022. CrI, credible interval; m, month.

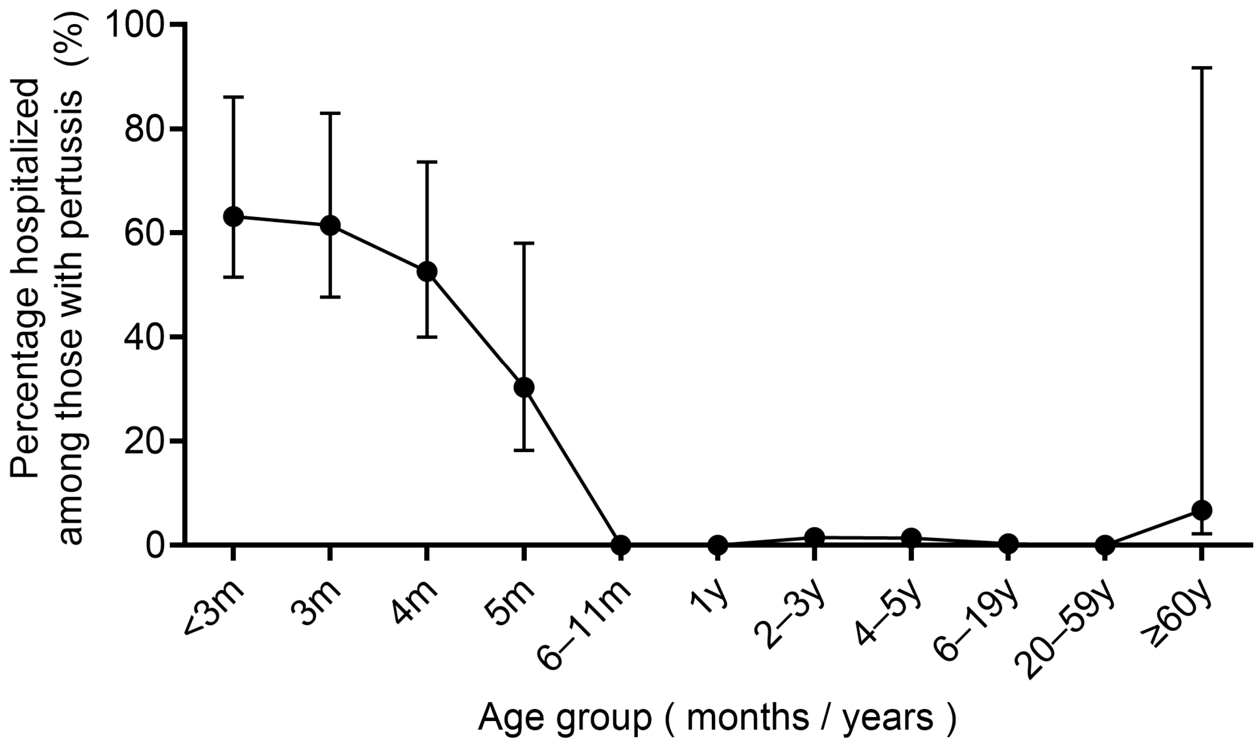


**Online Resource Figure S4.** Percentage hospitalized among those with pertussis (with 95%CrI) in Yiwu, Zhejiang province, from June 1, 2021, to May 31, 2022. CrI, credible interval; m, month; y, year.
